# Near‐Infrared‐Activated Thermochromic Perovskite Smart Windows

**DOI:** 10.1002/advs.202106090

**Published:** 2022-03-11

**Authors:** Sai Liu, Yang Li, Ying Wang, Kin Man Yu, Baoling Huang, Chi Yan Tso

**Affiliations:** ^1^ School of Energy and Environment City University of Hong Kong Tat Chee Avenue Kowloon Tong Hong Kong HKG China; ^2^ Department of Mechanical and Aerospace Engineering The Hong Kong University of Science and Technology Clear Water Bay, Kowloon Hong Kong HKG China; ^3^ Department of Physics City University of Hong Kong Tat Chee Avenue, Kowloon Tong Hong Kong HKG China

**Keywords:** building energy, perovskites, photothermal effect, smart windows, thermochromism

## Abstract

Perovskite‐based thermochromic smart windows that can change color have attracted much interest. However, the high transition temperature (>45 °C in air) hinders their practical application. Herein, a near‐infrared (NIR) activated thermochromic perovskite window that enables reversible transition cycles at room temperature is proposed. Under natural sunlight (>700 W m^−2^), it efficiently harvests 78% NIR light to trigger the thermochromism of perovskites, blocking the heat gain from both the visible and NIR light. Meanwhile, it also exhibits a low mid‐infrared emissivity of <0.3, suppressing thermal radiation to the indoor environment. A field test demonstrates that this smart window can reduce the indoor temperature by 8 °C compared to a normal glass window at noon. The near‐room‐temperature color change, multispectral thermal management, outstanding energy‐saving ability, and climate adaptability, and solution‐based process of this window make it unique and promising for real applications.

## Graphical abstract

A self‐activated thermochromic perovskite smart window (T‐PCL window) is developed. This window can smartly harvest the near‐IR part of sunlight to trigger the thermochromism at room temperature. Simultaneously, the T‐PCL window demonstrates room‐temperature solar regulation ability for visible light, shielding effect for near‐IR, and low thermal radiation for mid‐infrared, showing a promising future for application on energy‐efficient buildings.

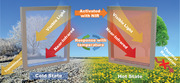

## 1 Introduction

To combat global warming, many governments of major economies have committed to reducing carbon emissions to net‐zero by the mid of this century.^[^ ^1^, ^2^ ^]^ It is reported that buildings are currently a major source of global carbon emissions,^[^ ^3^ ^]^ since more than 40% of energy consumed in cities is by buildings, especially their heating, ventilation, and air‐conditioning (HVAC) systems.^[^ ^4^, ^5^, ^6^, ^7^ ^]^ In buildings, windows are widely recognized as the least energy‐efficient components of the building envelope.^[^ ^8^ ^]^ Both their high optical transmittance and thermal conductivity lead to huge heat gain/loss. Therefore, energy‐efficient window technologies have been intensively developed over the past decades, such as the widely used low‐emissivity windows (Low‐E windows) and the emerging smart windows. Through switching between the transparent and opaque states, smart windows can intelligently regulate indoor solar irradiation in accordance with the ambient temperature and reduce energy consumption without compromising thermal comfort.^[^ ^9^ ^]^ According to the response to different external stimuli, smart windows can be categorized as electrochromic, photochromic, mechanochromic, thermochromic windows.^[^ ^5^ ^]^ Among them, thermochromic smart windows draw more and more attention due to their passive response (i.e., no need for external electricity, force, or light source) as they are more energy‐saving and adaptive to ambient temperature changes.^[^ ^10^, ^11^, ^12^ ^]^ To balance the viewing and energy‐saving performance, both the luminous transmittance (*τ*_lum_, i.e., total transmitted light of the window from wavelength of 380–780 nm) and solar modulation ability (Δ*τ*_sol_, i.e., the difference of solar transmittance *τ*_sol_ between the cold state and hot state of the thermochromic smart window) are important. And most importantly, smart windows must have a moderate transition temperature (*T*_c_) to trigger the thermochromic effect under ambient temperatures.^[^ ^13^, ^14^ ^]^

In recent years, a new type of thermochromic material, namely hybrid halide thermochromic perovskites (T‐Perovskite) was discovered. T‐Perovskites show promise for future smart window technologies due to their competitive optical performance as well as facile and inexpensive synthesis methods.^[^ ^15^, ^16^, ^17^, ^18^, ^19^, ^20^, ^21^, ^22^, ^23^, ^24^ ^]^ However, there is still an intractable issue impeding their room‐temperature applications. The *T*_c_ of reported T‐Perovskites to date varies from 35 to 105 °C and is much higher than comfortable room temperature (25 °C), which is also a common problem with most thermochromic materials.^[^ ^25^, ^26^, ^27^ ^]^ In 2015, Lin et al. published a pioneer work on the thermochromism of perovskites (CsPbI_3−_ *_x_* Br*_x_*, 0 ≤ *x* ≤ 3) and reported a high *T*_c_ at 105 °C.^[^ ^16^ ^]^ After that, Wheeler et al. proposed the methylammonium lead iodide (MAPbI_3_)‐methylamine (CH_3_NH_2_) complex to demonstrate a switchable T‐Perovskite with a *T*_c_ of 35 °C.^[^ ^19^ ^]^ Despite the reduced *T*_c_, the smart window must be tightly sealed in a toxic CH_3_NH_2_ atmosphere to trigger the thermochromic effect. More recently, Liu et al. developed a hydrated MAPbI_3−_ *_x_* Cl*_x_* (H‐MAPbI_3−_ *_x_* Cl*_x_*) T‐Perovskite that realized the thermochromism effect by dissociating and rebinding H_2_O from the MAPbI_3−_ *_x_* Cl*_x_* layer, without the need for a special gas atmosphere.^[^ ^17^ ^]^ However, to attain a *T*_c_ below 40 °C, the T‐Perovskite is required to be sealed in a double‐glazed window to strictly control the relative humidity (RH) in the gap at a level (RH <15%) far below that of the ambient environment, making assembly of the window difficult with risk of leakage during long‐term use. Therefore, developing alternative strategies to switch the T‐Perovskite at room temperature without the need to seal the material at a specific low humidity environment is urgent for the real application of T‐Perovskite smart windows.

One of the key features of T‐Perovskites is that their thermochromic effect occurs only in the visible and ultraviolet regions (300–780 nm).^[^ ^24^, ^28^ ^]^ In the near‐infrared region (NIR, 780–2500 nm) that accounts for 43% of solar irradiation, the T‐Perovskites have high transparency (>90%) at both cold and hot states.^[^ ^17^ ^]^ For this reason, the indoor environment is always heated by the transmitted NIR, which significantly impairs the thermal regulation effect of T‐Perovskites in the visible region. Herein, we propose an approach to realize room‐temperature transition in T‐Perovskites by taking advantage of this material deficiency. Specifically, the T‐Perovskite smart windows are coated with a photothermal material with high NIR absorption to heat the window surface by sunlight so that the transition temperature of the T‐Perovskite can be easily reached, triggering the thermochromism even at room temperature. Most importantly, it is not necessary to strictly control the humidity in the double‐glazed space of the T‐Perovskite smart windows, significantly increasing the ease with which the window can be assembled without the stringent sealing process. Meanwhile, due to the NIR shielding effect of the photothermal material, the solar heat gain of the indoor environment is remarkably reduced, further saving energy consumed by HVAC systems. To this end, cesium‐doped tungsten trioxide (CWO) was selected for both its high visible transmission (≈82%) and NIR absorption (≈90%), making it a perfect partner for T‐Perovskite smart windows that have high visible transmission (>85%) but low NIR absorption (<10%).^[^ ^17^, ^29^ ^]^

**Figure 1:**
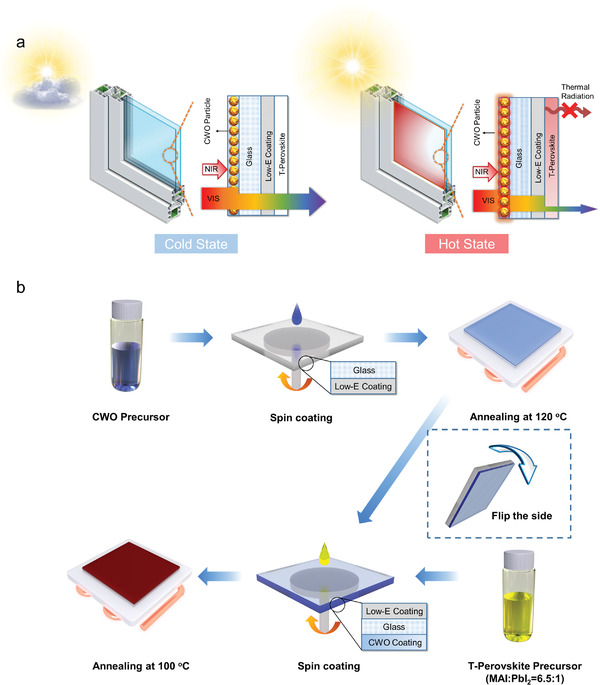
Fabrication and working principle of the T‐PCL window. a) Schematics showing the structure and the working principle of the T‐PCL window. b) Schematics of the solution‐based fabrication process of the T‐PCL window.

In this work, we fabricated a NIR‐activated thermochromic perovskite smart window (T‐PCL window, namely T‐Perovskite, CWO and Low‐e layer triple‐coated window) by integrating CWO with H‐MAPbI_3−_ *_x_* Cl*_x_* T‐Perovskite (**Figure** **1**a) and demonstrated its smart window performance. Under 0.7–1 sun illumination and at room temperature (23.5 °C), the CWO coating converted the NIR light into thermal energy and rapidly heated the window to 44–55 °C, successfully triggering the thermochromism of H‐MAPbI_3−_ *_x_* Cl*_x_*. In addition, to suppress the thermal radiation to the indoor environment, a low‐emissivity coating was used as the substrate of T‐Perovskite. In other words, the T‐PCL window can achieve thermal regulation in multiple spectral regions, including room‐temperature solar regulation ability for visible light, shielding effect for NIR, and low thermal radiation for mid‐infrared (MIR). Most importantly, a reversible thermochromic cycle of the T‐PCL window installed in a model house was successfully observed in a field test conducted in Hong Kong. This is the first work to realize the self‐activated thermochromism of the perovskite smart window under natural sunlight without the need for special environment (gas, ambient temperature, humidity, etc.) control. Besides, compared to a normal glass window, the T‐PCL window offered an 8.0 °C indoor temperature reduction at noon, verifying the high energy‐saving potential in practical applications.

## 2 Results and Discussions

### 2.1 Design and Fabrication of the T‐PCL Window

Figure 1a shows the 3D schematic of the proposed T‐PCL window, where the CWO layer faces the outdoor side and the T‐perovskite layer faces the indoor side. Considering the harsh outdoor environment and cleaning requirements, the CWO nanoparticles were embedded in acrylic resin to improve the anti‐abrasion ability. The CWO nanoparticles demonstrate high visible transmittance (≈80%) and efficient NIR absorption ability (≈90%) induced by the strong plasmon resonances and polaron excitation, acting as the solar‐thermal medium in the window.^[^ ^30^, ^31^ ^]^ Moreover, a most commonly used Low‐E coating (i.e., SnO_2_‐Ag‐SnO_2_ structure^[^ ^32^ ^]^) with high visible transparency (≈85%) (Figure S1, Supporting Information) was applied on the inner surface of the glass. The T‐Perovskite offers a color switchable function for the whole window. When the solar radiation is weak and/or the ambient temperature is low (e.g., morning and dusk), the absorbed NIR light by the CWO layer is not strong enough to elevate the window temperature above the *T*_c_ of T‐Perovskite. Therefore, the thermochromism is not activated and the window stays in the high‐transparency state (i.e., cold state) to maintain indoor lighting. In contrast, if the solar radiation is intense and/or the ambient temperature is high (e.g., noon and afternoon), the CWO layer can harvest more NIR photons, allowing the window temperature to rapidly reach the *T*_c_ to trigger the thermochromism. Then, the T‐PCL window switches to the colored state (i.e., hot state), shielding the solar radiation in both visible and NIR regions and considerably reducing indoor solar heat gain. In addition, the Low‐E layer not only protects the indoor environment from the high temperature of the window but also reduces the radiative heat loss of the T‐PCL window. In this way, the T‐PCL window can modulate the visible part of solar radiation between cold and hot weathers, constantly shielding most of the NIR and emitting less MIR, eventually achieving energy saving in multiple spectral regions.

To fabricate the T‐PCL window, a facile solution‐based method was employed. The prepared CWO solution was coated on the glass. XRD and TEM characterizations were used to examine its crystal structure and particle size. As shown in Figure S2 in the Supporting Information, the XRD diffraction peaks of the CWO agree with the standard pattern of Cs_0.33_WO_3_ (i.e., JCPDS No. 83‐1334), proving the purity of CWO nanoparticles. TEM image (Figure S3, Supporting Information) shows that the particle size of CWO is between 7 and 20 nm. A 4 mm thick Low‐E soda‐lime glass was selected as the substrate to mimic a real window. As shown in Figure 1b, the CWO‐acrylic resin solution was firstly spin‐coated on one side of the glass substrate and annealed to solidify the coating. A cross‐section SEM image of the CWO‐acrylic layer is shown in Figure S4a in the Supporting Information, and the corresponding elemental mapping measurement for tungsten (W) using Energy‐dispersive X‐ray spectroscopy (EDS) is shown in Figure S4b in the Supporting Information. It can be seen that W demonstrates identical intensity and continuous distribution throughout the film, manifesting uniform distribution of the CWO particles in the acrylic resin. The hydrated MAPbI_3−_ *_x_* Cl*_x_* (H‐MAPbI_3−_ *_x_* Cl*_x_*) T‐Perovskite developed by our group was selected as the thermochromic material.^[^ ^17^ ^]^ The H‐MAPbI_3−_ *_x_* Cl*_x_* demonstrates a bandgap transition (Figure S5, Supporting Information) at the cold (i.e., 3.13 eV) and hot states (i.e., 1.85 eV), leading to an obvious thermochromic effect in the visible range. The H‐MAPbI_3−_ *_x_* Cl*_x_* T‐Perovskite was spin‐coated on the other side of the glass (Low‐E side) with a solution of MAI and PbCl_2_ in the molar ratio of 6.5:1. The surface SEM image in Figure S6 in the Supporting Information confirms that the T‐Perovskite was uniformly coated on the Low‐E layer.

### 2.2 Optical Properties of the T‐PCL Smart Window

**Figure 2:**
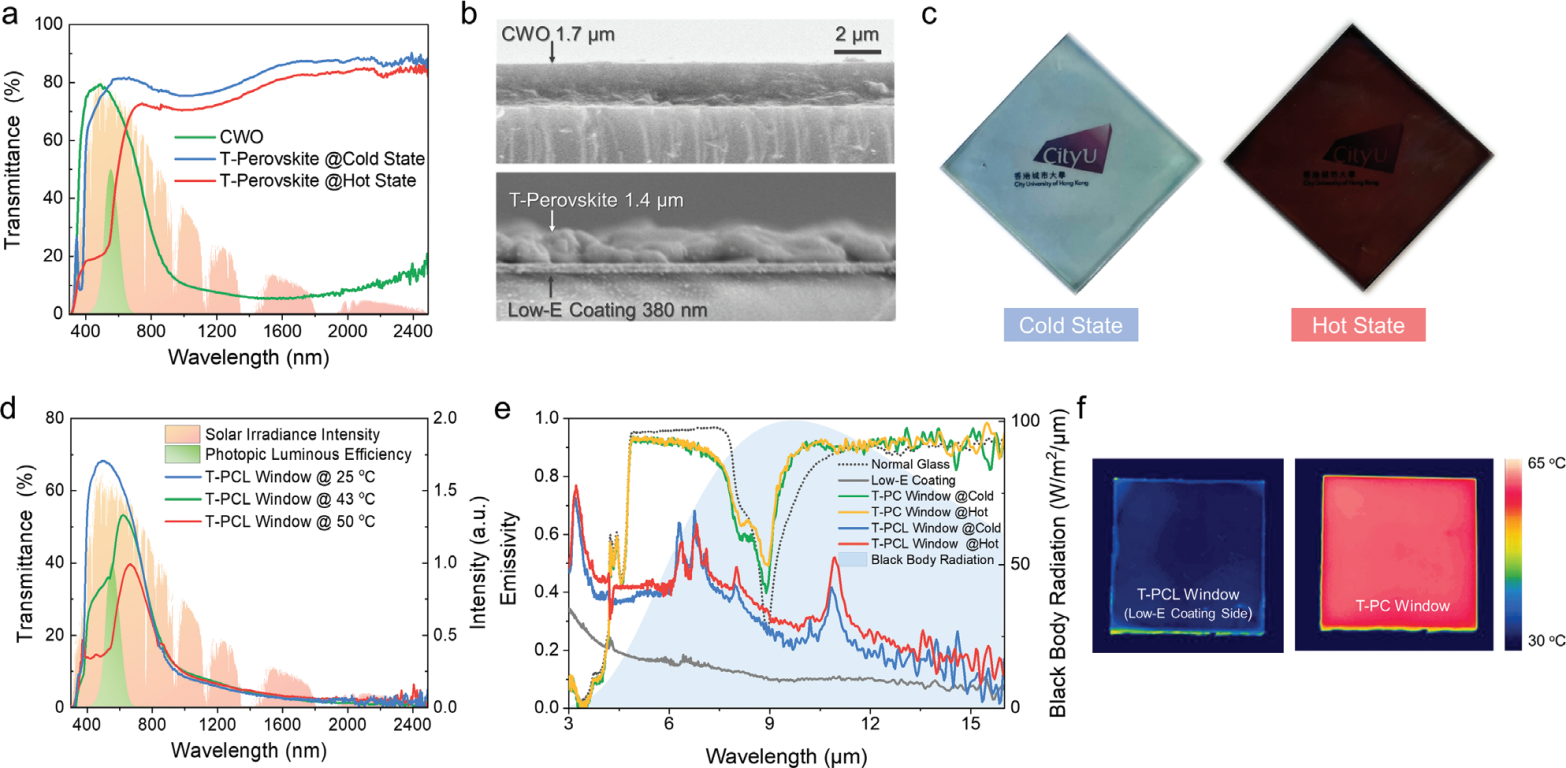
Optical Characterizations of the T‐PCL window. a) Transmittance spectra of the T‐Perovskite and CWO, together with the AM1.5 G solar spectrum. b) Cross‐sectional SEM images of the T‐PCL window. c) Photographs of the T‐PCL window at the cold and hot states. d) Transmittance spectra of the T‐PCL window at the cold state (25 °C), intermediate state (43 °C) and hot state (50 °C), together with the AM1.5 G solar spectrum. e) Emissivity spectra of various windows in the MIR region, together with the black body radiation spectrum at 25 °C. f) IR photographs of the T‐PCL window and T‐PC window on a hot plate at 60 °C.

**Figure** **2**a demonstrates the transmittance spectra of the single layer T‐Perovskite and CWO. An obvious transmittance contrast of T‐Perovskite in the visible light range between the cold state and hot state contributes to the significant solar modulation ability of the T‐PCL window, meanwhile, the constant NIR blocking of the T‐PCL window is attributed to the low transmittance of CWO in the NIR range. The SEM cross‐sectional image in Figure 2b shows that the CWO, T‐Perovskite and Low‐E coating can be uniformly coated on the glass substrate with thicknesses of 1.7, 1.4, and 0.38 µm, respectively. The fabricated T‐PCL smart window exhibits an obvious color switch with the temperature change due to the phase change of the T‐perovskite. Specifically, the thermochromic perovskite utilizes thermal heating to dissociate H_2_O from the MAPbI_3−_ *_x_* Cl*_x_* layer, thus transforming from the transparent state to the colored state. Therefore, in Figure 2c, at the cold state, the T‐perovskite exists in the transparent dihydrate form of MAPbI_6−*x*_Cl_*x*_·2H_2_O, and the T‐PCL window exhibits high transparency with a light blue color from the CWO layer, thus the logo below can be clearly seen. In contrast, at the hot state, the hydrated water of MAPbI_6−*x*_Cl*_x_* ·2H_2_O is released and the T‐Perovskite decomposes to the reddish MAPbI_3−_ *_x_* Cl*_x_* phase, leading to the dark reddish‐brown appearance, making the logo invisible, as shown in Figure 2c.^[^ ^17^ ^]^ Figure 2d compares the transmittance spectra change of the T‐PCL window from 25 °C (cold state) to 43 °C (intermediate state) and finally reaching 50 °C (hot state) with the solar irradiation intensity and it demonstrates that the T‐PCL window exhibits an exceptional spectral selectivity. Due to the high transmittance of the CWO, T‐perovskite, and Low‐E coating (Figure S1, Supporting Information; Figure 2a; Table S1, Supporting Information) in the photopic luminous efficiency dense region (380–780 nm), the T‐PCL window exhibits a high *τ*_lum_ of 65.7% at the cold state (25 °C). In addition, the NIR absorptance (*A*_NIR_, 780–2500 nm) of the T‐PCL smart window with CWO nanoparticles can reach 78.2%. When the window transitions to the hot state (50 °C), the T‐Perovskite absorbs the visible light, resulting in the reduction of *τ*_lum_ to 25.6%. Therefore, a large solar modulation is observed from 380 to 780 nm where the solar radiation is the strongest, yielding a high Δ*τ*_sol_ of 17.5%. The key optical properties of the T‐PCL window are comparable to those of the widely investigated VO_2_ thermochromic smart windows (e.g., *τ*_lum_ and Δ*τ*_sol_ are typically ≈50% and ≈10%, respectively^[^ ^33^, ^34^ ^]^). Moreover, the T‐PCL window can also effectively block NIR radiation, which contributes additional energy‐saving besides the thermochromic effect, compared with traditional thermochromic perovskite smart windows.

In addition to the solar modulation ability, the emissivity (*ε*) of a window is also critical for saving energy. Figure 2e shows the wavelength‐dependence emission curves of four different windows in the MIR region (3–16 µm): normal glass, Low‐E glass, T‐Perovskite and CWO dual‐coated normal glass (denoted as T‐PC window), and the T‐PCL window. The *ε* of the normal glass window and the Low‐E window are 0.84 and 0.1, respectively. The T‐PC window shows a similar emissivity curve as the normal window at both cold and hot states, resulting in a *ε* of 0.85. In comparison, with the addition of the Low‐E coating, the T‐PCL window offers a much lower *ε* of 0.27 at the cold state and 0.30 at the hot state. To visually demonstrate the emissivity difference, the T‐PC and T‐PCL windows were placed on a hot plate with a constant temperature of 60 °C and characterized by an IR camera (the default emissivity was set as 0.85). The T‐PCL window showed a much lower apparent temperature (35 °C) than the T‐PC window (60 °C) in the IR image (Figure 2f), which revealed the weaker thermal radiation and lower *ε* of the T‐PCL window. Compared to the T‐PC window, the T‐PCL window can suppress the radiative heat transfer between the window and the indoor environment, bringing at least two advantages. By installing a T‐PCL window, the room temperature will not be increased by the hot window through thermal radiation. Also, the T‐PCL window itself has less radiative heat loss than the T‐PC window, which helps it reach the transition temperature *T*_c_ faster under the same solar irradiation or even reach the *T*_c_ under weaker solar irradiation. This is highly favorable for thermochromic smart windows. The details are experimentally demonstrated and discussed in the next section.

### 2.3 Room‐Temperature Thermochromism of the T‐PCL Smart Window

A moderate transition temperature (*T*_c_) is vital for thermochromic smart windows. As the T‐Perovskite was deposited on the Low‐E coating in the T‐PCL window, it is necessary to identify whether the Low‐E coating affects the *T*_c_ of T‐Perovskite (H‐MAPbI_3−_ *_x_* Cl*_x_*) during the heating (*T*_c,h_) and cooling cycles (*T*_c,c_). Therefore, the *T*_c_ of the T‐PC window and the T‐PCL window were compared. As shown in Figure S7 in the Supporting Information, the transition processes of the two windows are almost identical. The calculated results (calculation method is in the Characterization section) show that the *T*_c,h_ and *T*_c,c_ of the T‐PC window are 42.6 and 36.1 °C, respectively, while those of the T‐PCL window are 42.4 and 35.3 °C, indicating that the Low‐E coating has no influence on the *T*_c_ of T‐Perovskite.

**Figure 3:**
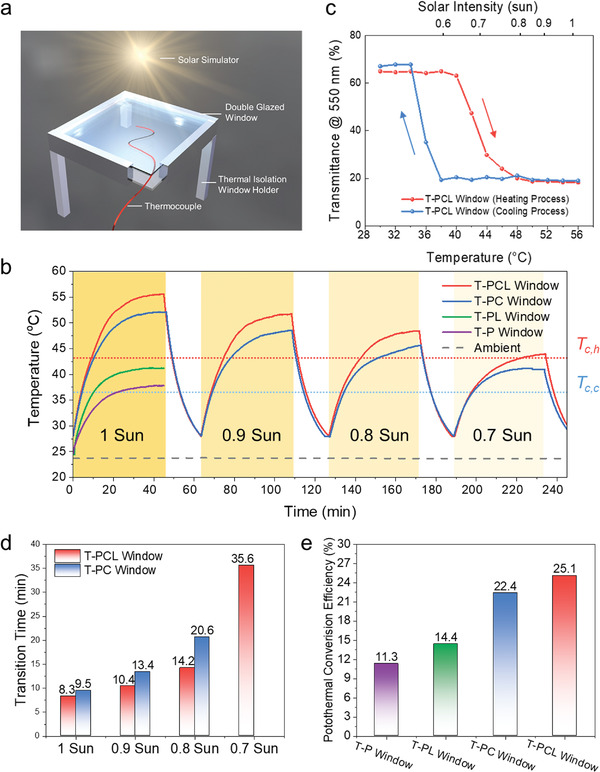
Photothermal performance of the T‐PCL window. a) Schematics of the experimental setup under a solar simulator for monitoring the window temperatures. b) Temperature‐response curves of the T‐P, T‐PL, T‐PC, T‐PCL windows under 0.7–1 sun illumination. The air temperature is 23.5 °C. c) The optical transmittance of the T‐PCL window at 550 nm as a function of temperature/solar intensity upon heating and cooling processes. d) Transition times of the T‐PC and T‐PCL windows under 0.7–1 sun. e) Photothermal conversion efficiency of the T‐P, T‐PL, T‐PC, and T‐PCL windows.

To evaluate the photothermal response and verify the effectiveness of the T‐PCL window under solar irradiation, the T‐PCL window as well as three control samples were placed under a solar simulator to record the temperature response curves (the indoor temperature is 23.5 °C). The three control windows were the T‐perovskite coated normal glass (T‐P window), T‐perovskite coated Low‐E glass (T‐PL window), and T‐Perovskite and CWO dual‐coated normal glass (T‐PC window) (Figure S8, Supporting Information). To mimic the real application scenario, a double‐glazed structure was used in this test to protect the T‐Perovskite layer, where the coated glass was on the top and a normal glass was placed at the bottom. A thermocouple was attached to the T‐Perovskite layer to monitor the surface temperature of each window (**Figure** **3**a). As shown in Figure 3b, under 1 sun illumination, the steady‐state temperatures of both T‐P and T‐PL windows without a CWO layer were only 37.8 and 41.1 °C, respectively, which were below the *T*_c,h_ (42.6 °C). Although the *T*_c_ of H‐MAPbI_3−_ *_x_* Cl*_x_* is already much lower compared with other reported T‐Perovskites,^[^ ^16^, ^18^, ^24^ ^]^ it remains challenging to activate the color change of H‐MAPbI_3−_ *_x_* Cl*_x_* even under 1‐sun irradiation (the maximum natural sunlight). In contrast, the temperatures of both T‐PC and T‐PCL windows rapidly exceeded the *T*_c,h_ of T‐Perovskite within 10 min, and reached a steady‐state of 52.1 and 55.6 °C, respectively. These results strongly suggest the effectiveness of the CWO layer in efficient photothermal conversion. It is also found that the steady‐state temperature of the T‐PCL window with a Low‐E layer was 3.5 °C higher than that of the T‐PC window, and the steady‐state temperature of the T‐PL window was 3.4 °C higher than that of the T‐P window. These phenomena confirm that the Low‐E layer enables the window to reach a higher temperature and trigger the phase transition of the T‐Perovskite faster. Except at a tropical summer noon, the solar irradiation during day time around the world is normally lower than the maximum value of 1000 W m^−2^ (1 sun AMO). Therefore, testing the applicability of the T‐PCL window operating under strong but not maximum solar irradiation is of practical importance. As shown in Figure 3b, both the T‐PCL and T‐PC windows were able to reach the *T*_c,h_ of the T‐perovskite under 0.9 and 0.8 sun solar irradiances. Moreover, under 0.7‐sun illumination, the T‐PC window cannot activate its thermochromic effect, while the thermochromism of the T‐PCL window can be maintained, suggesting a broader applicable time range in a day. When the ambient temperature is around 23.5 °C, such a smart color switch under (or above) 0.7 sun is highly favorable for real‐world applications since it perfectly covers those hot hours during the daytime. Moreover, with the increase/decrease of the ambient temperature, this automatic color switch will be activated under weaker/stronger solar radiation, indicating its great adaptability to diverse climates. Additionally, we also examined the thermochromic effect of the T‐PCL window in two different weather conditions (e.g., very cold and dry as well as hot and humid weather conditions), and it was found that the T‐PCL window also demonstrated a good adaptability (Note S1 and Figure S9, Supporting Information). Furthermore, to quantitatively evaluate the transmittance of the T‐PCL window under different solar intensity levels, we firstly measured the transmittance of the T‐PCL windows as a function of temperature at the wavelength of 550 nm (Figure 3c). At the same time, based on the temperature‐response curves of the T‐PCL windows under 0.7–1 sun illumination (Figure 3b), the corresponding window temperature can be obtained in different solar intensity levels. Then, the solar intensity was marked on the top of the *x* axis in Figure 3c. From this figure, it can be seen that the transmittance of the T‐PCL window decreased from 65% to 20% (i.e., from the transparent state to the colored state) as the solar intensity increased, meaning the indoor lighting could be smartly modulated with the intensity of sunlight. Additionally, the window temperature increases most significantly from 0.7 to 0.8 sun. The reason is that at this intermediate stage, the T‐Perovskite layer gradually transfers from the transparent state to the colored state (i.e., reddish brown), thus parts of the visible light are also absorbed, besides the NIR heat absorbed by the CWO layer, further boosting the temperature increase of the T‐PCL window.

The transition time of thermochromic smart windows, defined as the time required to switch between the cold and hot states under a constant solar irradiance, is also an important parameter for their real applications. As shown in Figure 3d, both T‐PCL and T‐PC windows showed acceptable transition times, especially under strong solar irradiance. Under 1‐sun illumination, the transition times for T‐PCL and T‐PC windows were 8.3 and 9.5 min, respectively. The faster response time of the T‐PCL window can be attributed to its higher photothermal conversion efficiency enabled by the lower emissivity. To quantitatively compare the photothermal performance of different windows, their photothermal conversion efficiency (PCE), which is defined as the ratio of the energy for temperature increase inside the window to the incident solar irradiation,^[^ ^35^ ^]^ is shown in Figure 3e. Due to the collective effects of both the CWO coating and Low‐E coating, the PCE of the T‐PCL window can reach 25.1%, showing a huge improvement of 122.1% compared with that of the T‐P window (11.3%). In short, the self‐activation switch, wide applicable time range in a day, fast response, and great adaptability to diverse climates of the T‐PCL window make it an advanced smart window, which is highly attractive for practical applications worldwide.

### 2.4 Application of the T‐PCL Smart Window in the Real Environment

**Figure 4:**
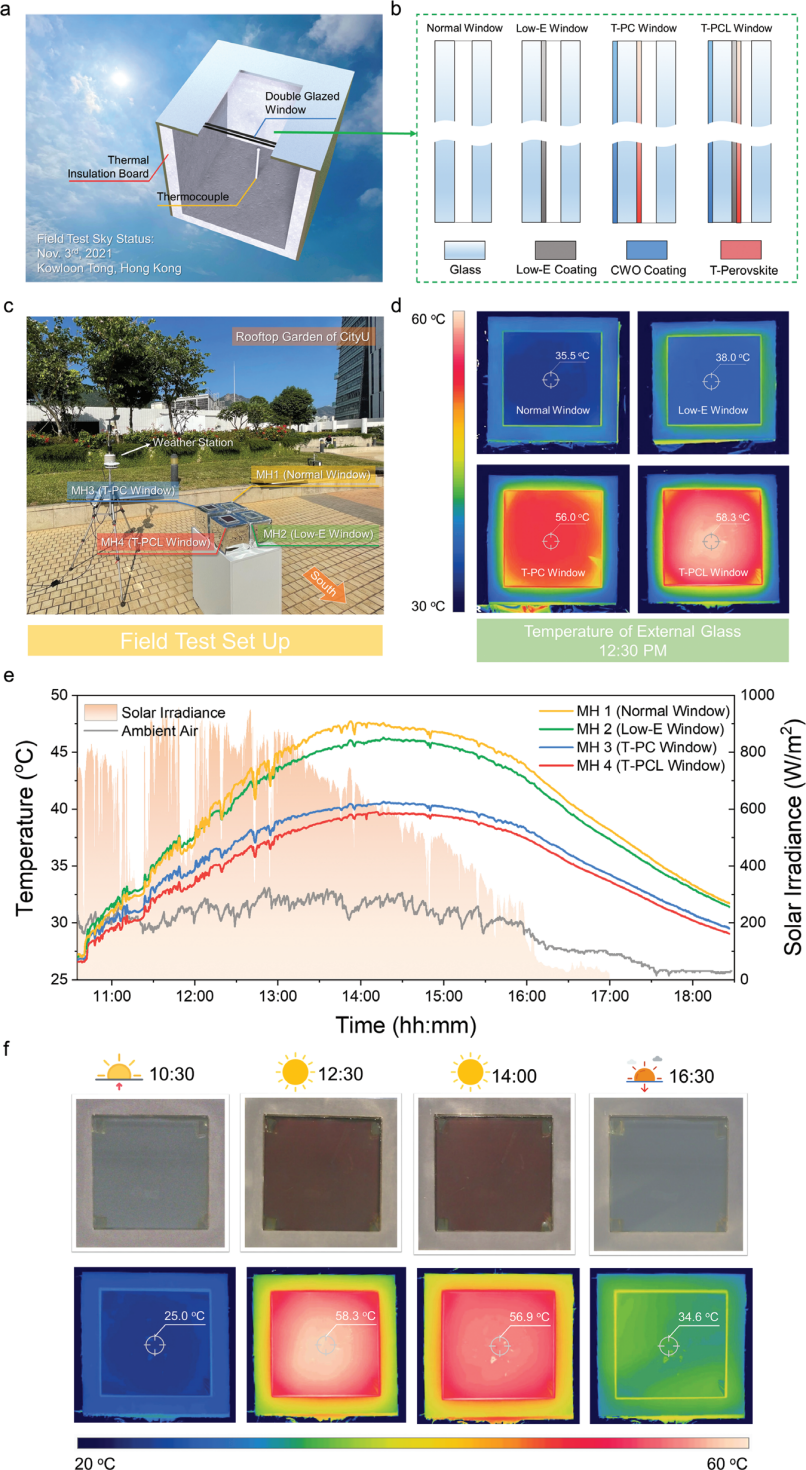
Model house field test. a) Schematics of the model house internal structure. b) Schematics of double‐glazed windows used for the model house field test. c) Experimental setup for the model house field test. d) Surface temperature of the windows applied on each model house taken by an IR imager. e) Indoor temperature curves for the model house field test on the 3^rd^ November, 2021 in Hong Kong. f) Reversible color and IR images of the T‐PCL window throughout the testing day.

To assess the thermochromic effect and the energy‐saving performance of the smart windows in real applications, model house field tests were conducted in Hong Kong's subtropical climate (the weather data monitored by the weather station are shown in Table S2 in the Supporting Information). As shown in **Figure** **4**a, the model houses were made of acrylic boxes with a dimension of 20 × 20 × 20 cm^3^ and sealed with thermal insulation materials. Indoor temperatures were monitored by thermocouples. In the field tests, four kinds of double‐glazed windows (9 × 9 cm^2^) were installed on four different model houses: a normal window on model house 1 (MH1), a Low‐E window on MH2, a T‐PC window on MH3, and a T‐PCL window on MH4 (Figure 4b). The experimental setup is shown in Figure 4c.

Figure 4d shows the surface temperature of each window captured by an IR camera at noontime when the solar irradiation reached a peak value of ≈900 W m^−2^. Owing to the lack of the CWO, the normal window and Low‐E window only reached 35.5 and 38.0 °C, respectively, which is below the *T*_c,h_ of the T‐Perovskite. By comparison, the T‐PC and T‐PCL windows reached much higher temperatures of 56.0 and 58.3 °C, respectively, successfully activating the thermochromic effect. Figure 4e shows the indoor temperature curves of the four model houses. The indoor temperature of MH1 with normal glass was the highest among the four houses during the whole day and reached a peak of 47.7 °C at around 14:00. The Low‐E coating of MH2 resulted in a 1.5 °C temperature reduction compared with MH1. In contrast, the peak indoor temperature of MH3 and MH4 were 40.7 and 39.7 °C, respectively, which were 7.0 and 8.0 °C lower than the MH1. These remarkable temperature reductions prove the great energy‐saving ability of the T‐PC and T‐PCL windows by utilizing the CWO layer to activate the thermochromic effect of the T‐Perovskite. In addition, the incorporation of a Low‐E layer in the T‐PCL window further helped in reducing the indoor temperature by 1 °C. It should be noted that the window temperatures of MH3 and MH4 were much higher than those of MH1 and MH2 (Figure 4d), although the indoor air temperatures of MH3 and MH4 were lower. This inconsistency implies that direct solar radiation dominates the heat gain of the indoor environment rather than the heat transfer between the window and the indoor air.

The entire thermochromic process of the T‐PCL window was also recorded during the field test and is shown in Figure 4f. At 10:30, the window is highly transparent with a surface temperature of 25 °C. With the increase of solar radiation and ambient temperature, the window temperature rose to above the *T*_c,h_, reaching 58.3 °C at 12:30 and 56.9 °C at 14:00. During these hot hours at noon and in the afternoon, the color change of T‐Perovskite was activated, thus effectively reducing heat gain. Later, the T‐PCL window switched back to the transparent state (window temperature of 34.6 °C at 16:30) due to the weaker solar intensity in the late afternoon. In practical applications, this regulation is highly smart because in the morning and late afternoon when the solar radiation is weak, the high transparency of the window can meet the indoor lighting demand. At the same time, there is still air‐conditioning demand, but the energy load of the HVAC system is not as high as noon and afternoon, so the energy‐saving function of the T‐PCL smart window only needs to be maintained by the NIR shielding of the CWO layer. While at noon and in the afternoon with the strong sunlight, the T‐PCL window transferred to the colored state to further reduce the energy load of HVAC systems by blocking sunlight.

To decouple the contribution of perovskites from CWO, we have also compared the indoor air temperature by installing the T‐PCL window and pure CWO window in two identical model houses, respectively. This experiment was conducted in an air enthalpy testing laboratory equipped with a solar simulator (Jockey Club Controlled Environment Test Facility, HKUST). This laboratory can accurately control the temperature and humidity within ±0.1 °C and ±3% RH, respectively. The ambient temperature of the laboratory was set at 32 °C, and the power of the solar simulator was set as 0.9 sun, which are consistent with field‐test day on Nov. 3^rd^, 2021 in Hong Kong. Figure S10a in the Supporting Information shows the experimental setup. Specifically, the CWO coated window was installed on a new model house 5 (MH5). To fairly compare the indoor air temperature of the MH4 (T‐PCL window) and MH5 (CWO window), the initial indoor air temperatures of these two model houses was 28 °C before exposure to sunlight from the solar simulator. Figure S10b in the Supporting Information shows the indoor air temperature curves of the two model houses. The indoor air temperature of MH4 with the T‐PCL window reached 38.2 °C after 75 min, while the indoor air temperature of MH5 with the CWO window was 3 °C higher than that of the MH4, reaching 41.2 °C. The lower temperature of MH4 was contributed by the thermochromic effect of the T‐PCL window at the visible light region. This result proves the significant energy saving ability of the T‐Perovskite layer in the T‐PCL window compared with the CWO coated window.

### 2.5 Energy‐Saving Potential Evaluation

**Figure 5:**
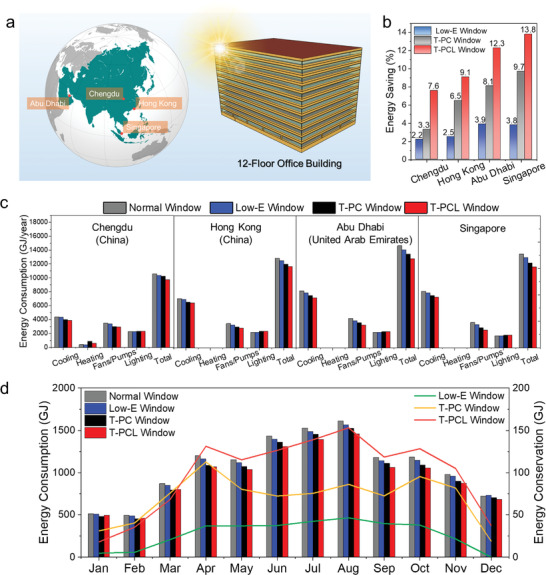
Simulation for energy‐saving performance in buildings. a) The geographic location of Chengdu, Hong Kong, Abu Dhabi, and Singapore as well as the building model used in the simulation. b) Total energy saving by using a Low‐E window, T‐PC window, and T‐PCL window over a normal window. c) Detailed energy consumption of cooling, heating, fans/pumps, and lighting in the building. d) Monthly energy consumption and conservation in Hong Kong.

To further evaluate the energy‐saving performance of the smart windows in actual‐size buildings, an Energy Plus program was conducted, where a 12‐floor reference office building (window to wall ratio: 37.5%) built by the U.S. Department of Energy (DOE) was used in the simulation.^[^ ^36^ ^]^ The building data are summarized in Table S3 in the Supporting Information, and the optical properties of the normal glass, Low‐E glass, T‐PC glass, and T‐PCL glass (Figure 4b) used in the simulation are given in Table S4 in the Supporting Information. To investigate the applicability of the T‐PCL window in different climate regions, four northern hemisphere cities were selected from the north to south (**Figure** **5**a), namely Chengdu (China), Abu Dhabi (UAE), Hong Kong (China), and Singapore. The climate information of these cities is shown in Table S5 in the Supporting Information.

The overall simulated energy‐saving potential by using different windows compared with the normal window is shown in Figure 5b. As expected, both the T‐PC window and T‐PCL window demonstrate better energy‐saving ability than the Low‐E glass window. In particular, the T‐PCL window can reduce 7.6%, 9.1%, 12.3%, and 13.8% total energy consumption (including HVAC system and lighting system) from Chengdu (temperate), Hong Kong (subtropical), Abu Dhabi (subtropical, desert) to Singapore (tropical), respectively. Clearly, the T‐PCL window is especially energy‐saving in subtropical and tropical regions, because the original huge energy used for the building cooling system and ancillary equipment (fans and pumps) can be significantly reduced (Figure 5c). Deploying the T‐PCL window technology in these areas can contribute more to the carbon‐neutrality for buildings. Both the smart optical regulation ability of T‐Perovskite, the strong NIR shielding of CWO, and the low emissivity coating contribute to this significant reduction. The energy‐saving performance over a year in the local city of Hong Kong was further investigated. The results shown in Figure 5d illustrate that both the monthly energy consumption and energy conservation by using the T‐PCL window reached their peaks in the summer, implying that the main contributor of building energy consumption in Hong Kong is space cooling, and the T‐PCL window is perfectly applicable to Hong Kong's climate.

### 2.6 Stability Test of the T‐PCL Window

**Figure 6:**
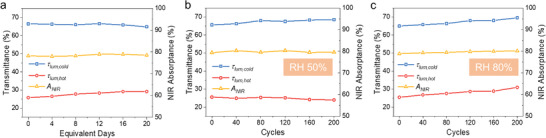
Stability test for the T‐PCL window. a) Stability test of the T‐PCL window using a solar simulator for 20 equivalent days. Optical performance (*τ*_lum,hot_ *, τ*_lum,cold_ and *A*_NIR_) of the T‐PCL window after 200 cycles under b) 50% RH and c) 80% RH.

The optical stability of the smart windows under sunlight and thermal cycles is necessary for their applications. Therefore, a durability test was conducted for the T‐PCL window. Firstly, the T‐PCL window was exposed under sunlight to examine its long‐term light resistance. To accelerate the aging test, we used a solar simulator with the setting power of 2 sun (2000 W m^−2^). According to the weather data from the Hong Kong Observatory, the daily global solar radiation throughout a year in Hong Kong is 13.23 MJ m^−2^.^[^ ^37^ ^]^ So, the T‐PCL window was exposed under the solar simulator for 36 hours, which is equivalent to 20 days in the real scenario. The T‐PCL window was cooled down to the transparent state after exposure under sunlight every 1.8 hours (equivalent to 1 day). **Figure** **6**a illustrates the stability test results, and it is found that the *A*_NIR_ remains almost unchanged after 20 equivalent days, showing a good durability of the CWO layer. Additionally, it can be observed that the *τ*_lum,cold_ of the T‐PCL window is relatively stable, while the *τ*_lum,hot_ increases from 25.8% to 29.2%. However, it should be noted that these results were obtained under 2 sun solar radiation, so it is expected that durability could be further extended under a lower solar intensity. Furthermore, the stability of the T‐PCL window after thermochromic transition cycles at different relative humidity (RH) levels were also investigated. The cooling and heating cycle test was conducted at the air enthalpy testing laboratory. 50% RH (moderate humidity) and 80% RH (high humidity) conditions were considered in the cycle test. Figure 6b illustrates that no significant change can be seen over the 200 cycles for the *τ*_lum_ and *A*_NIR_ under 50% RH. This result demonstrates that the reversible transition of the T‐PCL window can be repeated with stable performance under a moderate humidity condition. For the condition of 80% RH (Figure 6c), the *A*_NIR_ of the T‐PCL window remains stable after 200 cycles, proving that the CWO layer was not influenced by the humidity. However, the increase in *τ*_lum_ at both the cold and hot states could be observed, which may be caused by the detrimental effect of the high humidity on the thermochromic perovskite. In practical applications, the humidity in our ambient environment normally lies in the moderate level, implying that the lifetime of the T‐PCL window is expected to be further extended.

## 3 Conclusion

In this study, a NIR‐activated perovskite thermochromic smart window (T‐PCL window) that can achieve energy saving in multiple spectral regions was first explored. The T‐PCL window can selectively absorb NIR to generate heat and activate the thermochromism of T‐Perovskite, which smartly modulates the solar heat gain from the window. This high‐level smart window demonstrates reversible color change with a transmittance of *τ*_lum_ = 65.7% and 25.6% at the cold and hot states, smart solar modulation ability of Δ*τ*_sol_ = 17.5%, strong near‐infrared absorption of *A*_NIR_ = 78.2%, and low emissivity of *ε* < 0.3. Importantly, for the first time, under natural sunlight without any atmosphere control strategies, a self‐activated reversible thermochromic cycle was successfully observed in the field test, which is highly desired in practical applications. Additionally, a large 8.0 °C indoor temperature reduction was achieved by using the T‐PCL window, demonstrating its great energy‐saving potential. The Energy Plus simulation also reveals the promising energy conservation of 9.1%‐13.8% by applying the T‐PCL window technology in subtropical and tropical regions. However, it should be pointed out that because the CWO layer in the T‐PCL window constantly blocks over 70% NIR light of the solar radiation, the indoor heating ability by sunlight in the cold seasons will be influenced. Therefore, a thermochromic perovskite smart window with dynamically tunable solar transmittance in both visible and NIR regions is a potential future research direction. Additionally, the advanced strategies, such as compositional engineering, morphology engineering and solvent engineering, to further improve the stability of the thermochromic perovskite under a high humidity condition also deserves to be studied in the future work. In summary, this study presents a smart method to self‐activate the thermochromic perovskite smart window at room temperature and to further enhance its energy‐saving performance, representing a major step towards practical application of thermochromic smart windows.

## 4 Experimental Section

### Materials and Chemicals

The CWO nanoparticles solution (2‐ethoxyethanol as the solvent) and acrylic resin were supplied by TPK Materials Solution. The CH_3_NH_3_I (MAI, 99.5%) was purchased from Xi'an Polymer Light Technology. PbCl_2_ (99%) and PbI_2_ (99%) were provided by Sigma‐Aldrich. Dimethylformamide (DMF, ≥99.5%) was purchased from Alfa Aesar. The normal glass and Low‐E glass were provided by Ruihong Glass Ins.

### Fabrication of Smart Windows

The glass substrates were cleaned with detergent, ethanol, and deionized (DI) water in an ultrasonic bath for 15 min respectively, followed by drying with N_2_. Both sides of the glass substrates were further cleaned in a UV ozone cleaner for 15 min. Two precursors, namely CWO precursor and thermochromic perovskite H‐MAPbI_3−_ *_x_* Cl*_x_* precursor were used in this work. To prepare the CWO precursor, the CWO nanoparticle solution was mixed with the acrylic resin in the weight ratio of 7:3, followed by stirring for 15 min. The H‐MAPbI_3−_ *_x_* Cl*_x_* precursor was synthesized in a glovebox (water content ≤ 1 ppm, oxygen content ≤ 1 ppm). MAI and PbCl_2_ were mixed in the molar ratio of 6.5:1 and dissolved in DMF, followed by stirring at 50 °C for 1 hour. Four types of windows were prepared, namely T‐P, T‐PL, T‐PC, and T‐PCL windows (Figure S8, Supporting Information), and each layer was spin‐coated following the sequence shown in Figure S8 in the Supporting Information. Specifically, CWO was spin‐coated on the normal glass or the Low‐E glass at 1000 rpm for 30 s, and the sample was annealed at 120 °C for 5 min. Then the sample was moved to the glovebox to deposit the T‐Perovskite on the other side. The H‐MAPbI_3−_ *_x_* Cl*_x_* precursor was spin‐coated at 2000 rpm for 30 s, followed by annealing at 100 °C for 1 hour to evaporate the residual DMF. To assemble the double‐glazed window for the field test, the edges of the two glass panes were sealed using spacer tape (3M VHB Tapes). The double‐glazed window was then embedded in the frame of the model house. The detailed configuration of each double‐glazed window is shown in Figure 4b.

### Characterization

The CWO solution was spin‐coated on the quartz glass to characterize the crystalline structure by X‐ray diffraction (XRD) (PANalytical X'Pert3 powder diffractometer). The TEM (2010F, Jeol) was used to characterize the size of the CWO nanoparticles. The cross‐sectional SEM image of the T‐PCL window was obtained by a ZEISS EVO MA10 SEM. The transmittance spectrum was obtained by a UV‐Vis‐NIR spectrophotometer from 300 to 2500 nm (Lambda 950, Perkin Elmer equipped with a 150 mm integrating sphere detector). A temperature controller (including a heater, a T‐type thermocouple, and a Digi‐sense TC9600 temperature controller) was attached to control the sample temperature while measuring the transmission at both cold (25 °C) and hot states (50 °C). To quantify the amount of transmitted visible light, the luminous transmittance (*τ*_lum_) of each window was calculated by ( $\tau_{\mathrm{lum}}=\;\frac{\int_{\lambda \;=\;380\mathrm{nm}}^{780\mathrm{nm}}\bar{y}\left(\lambda \right)\tau \left(\lambda \right)d\lambda }{\int_{\lambda \;=\;380\mathrm{nm}}^{780\mathrm{nm}}\bar{y}\left(\lambda \right)d\lambda }$), where *τ(λ)* is the transmittance of the windows at a wavelength *λ*. *y ®(λ)* is the photopic luminous efficiency of the human eye defined by the CIE (International Commission on Illumination) standard. And the total solar transmittance is defined as transmittance *τ*_sol_ ( $\tau_{sol}=\;\frac{\int_{\lambda \;=\;300\mathrm{nm}}^{2500\mathrm{nm}}AM_{1.5}\left(\lambda \right)\tau \left(\lambda \right)d\lambda }{\int_{\lambda \;=\;300\mathrm{nm}}^{2500\mathrm{nm}}AM_{1.5}\left(\lambda \right)d\lambda }$), where *AM*_1.5_ *(λ)* is the AM1.5 G solar irradiance spectrum. The Δ*τ*_sol_ could then be calculated as $\Delta \;\tau_{sol}=\;\tau_{\mathrm{sol}}^{\mathrm{cold}}-\tau_{\mathrm{sol}}^{\mathrm{hot}}$. The NIR absorption was calculated from the range of 780–2500 nm and defined as $A_{\mathrm{NIR}}=\;\frac{\int_{\lambda \;=\;780m}^{2500\mathrm{nm}}AM_{1.5}\left(\lambda \right)A\left(\lambda \right)d\lambda }{\int_{\lambda \;=\;780\mathrm{nm}}^{2500\mathrm{nm}}AM_{1.5}\left(\lambda \right)d\lambda }$, where *A(λ)* is the spectral absorption. To measure the transition temperature *T*_c_ of the T‐Perovskite, a heating and cooling cycle for the samples was conducted on a high‐precision temperature controlled hot plate (CHEMAT 4AH) between room temperature and 60 °C, at intervals of 2 °C. For each temperature set point, the samples were kept on the hot plate for 2 min to ensure the stability of the color. At the same time, the transmittance at 550 nm was measured by a Lens Transmission meter (SDR8508). Then, the *T*_c_ can be calculated by plotting the first derivative of the transmittance to the temperature as a function of temperature, where *T*_c_ is the minimum value point at the first derivative. The thermal response test was conducted under a solar simulator (SCIENCETECH‐300, Class AAA), the power was tuned between 0.7 sun and 1 sun. A T‐type thermocouple was used to measure the sample temperature, and the data was recorded by NI 9213 (National Instrument). The photothermal conversion efficiency of the window was calculated as *η =* $\frac{c_{g}m_{g}\Delta T_{g}}{IA\Delta t}\;$, where *c*_g_ is the heat capacity and *m*_g_ is the mass of the glass, Δ*T*_g_ is the temperature difference between the ambient air and the window, *I* is the solar power intensity, *A* is the area of the window surface, and Δ*t* is the temperature increase time. The surface temperature of windows in the field test was captured by an IR camera (FLIR E75).

## Conflict of Interest

The authors declare no conflict of interest.

## Supporting information

Supporting information

## Data Availability

The data that support the findings of this study are available in the supplementary material of this article.
