# Supplementary material for: Near‐Infrared‐Activated Thermochromic Perovskite Smart Windows
Source: Adv Sci (Weinh). 2022 Mar 11;9(14):2106090. doi: 10.1002/advs.202106090 (PMC9108621; doi:10.1002/advs.202106090)
Supplement: Supplementary file 1 — Supporting information [file ADVS-9-2106090-s001.pdf]

ADVANCED SCIENCE
Open Access

Supporting Information

for *Adv. Sci.*, DOI 10.1002/advs.202106090

Near-Infrared-Activated Thermochromic Perovskite Smart Windows

*Sai Liu, Yang Li, Ying Wang, Kin Man Yu, Baoling Huang and Chi Yan Tso**

Supporting Information

Near-Infrared-Activated Thermochromic Perovskite Smart Windows

Sai LIU[1,4], Yang LI[2,4], Ying WANG[3], Kin Man YU[3], Baoling HUANG[2] and Chi Yan TSO[1,*]

[1]School of Energy and Environment, City University of Hong Kong, Tat Chee Avenue Kowloon Tong, Hong Kong, China.

[2]Department of Mechanical and Aerospace Engineering, The Hong Kong University of Science and Technology, Clear Water Bay, Kowloon, Hong Kong, China.

[3]Department of Physics, City University of Hong Kong, Tat Chee Avenue, Kowloon Tong, Hong Kong, China.

[4]These authors contributed equally.

*E-mail: chiytso@cityu.edu.hk (C.T.)

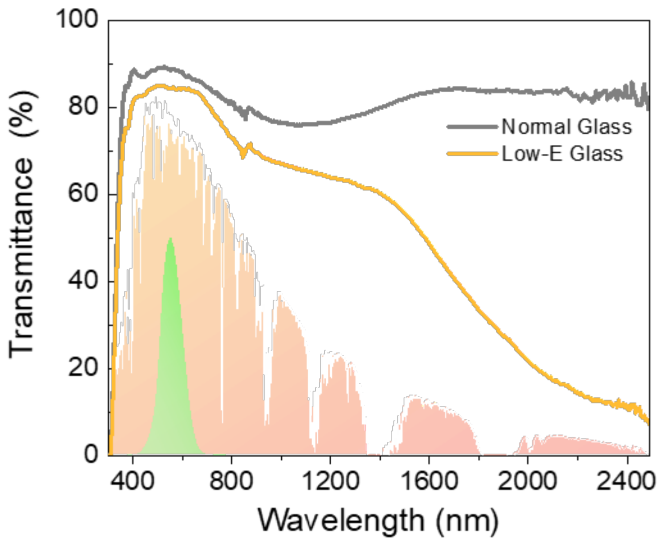

Figure S1. Transmittance spectrum of normal glass and Low-E glass. The green area is the photopic luminous efficiency of the human eye defined by the CIE (International Commission on Illumination) standard. The orange area is the solar irradiance spectrum for an absolute air mass of 1.5.

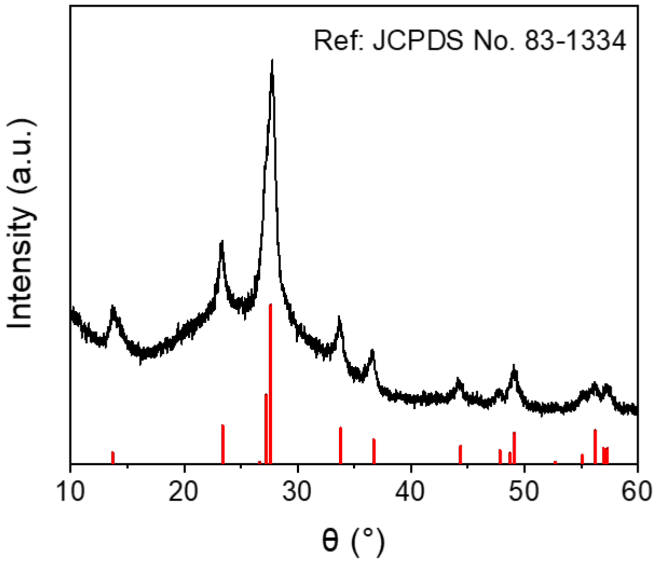

Figure S2. XRD pattern of CWO and the reference pattern of JCPDS No. 83-1334.

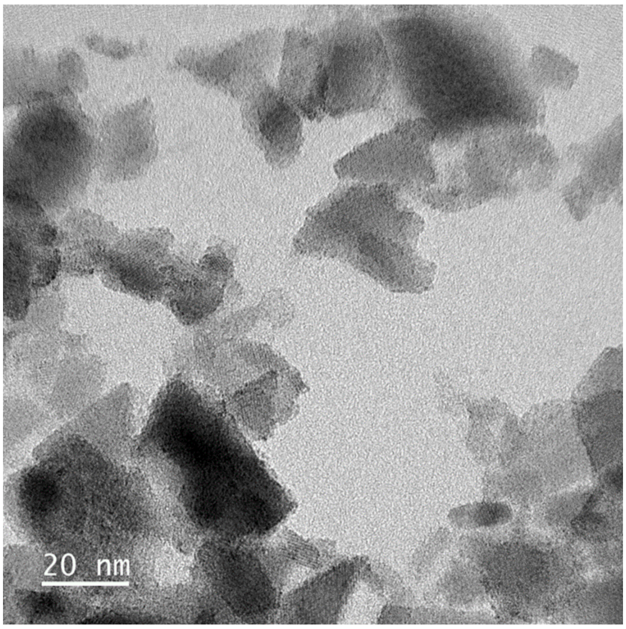

Figure S3. TEM image of CWO particles.

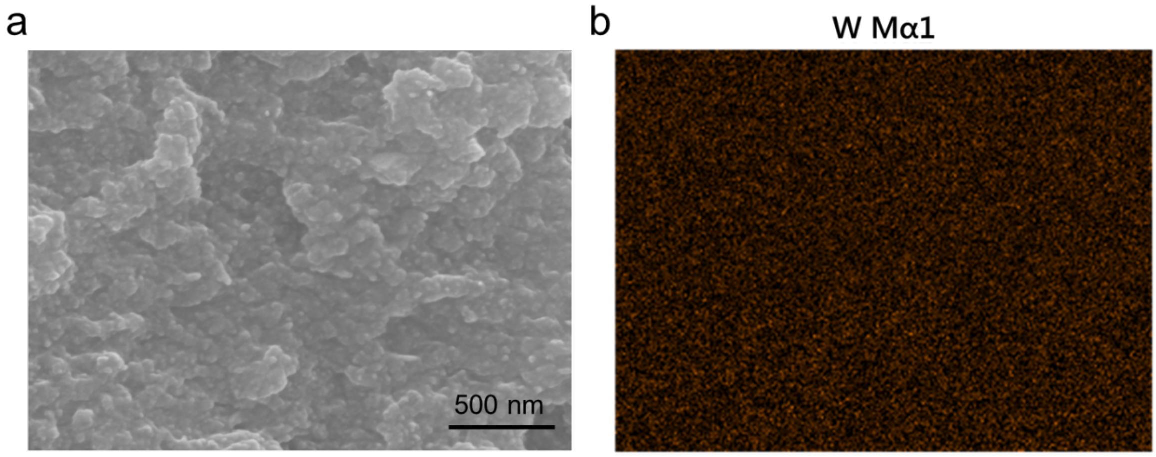

Figure S4. (a) Cross-section SEM image of the CWO-acrylic resin layer and corresponding (b) EDX mapping of tungsten.

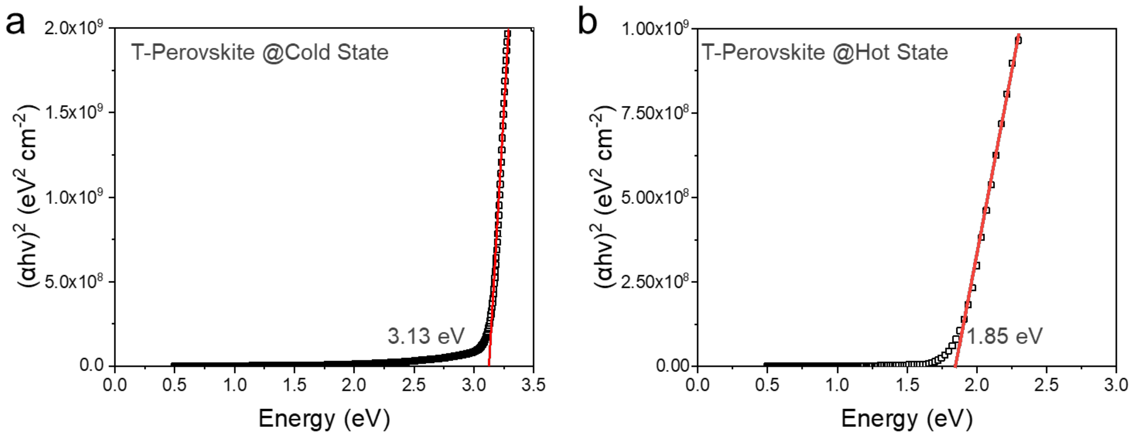

Figure S5. Band gap of T-Perovskite (H-$MAPbI_{3-x}Cl_x$) at the cold and hot states.

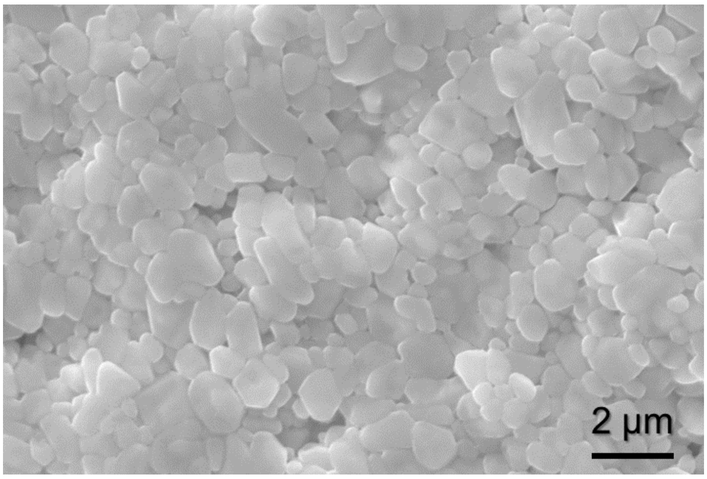

Figure S6. SEM image of the T-Perovskite film coated on the Low-E glass substrate.

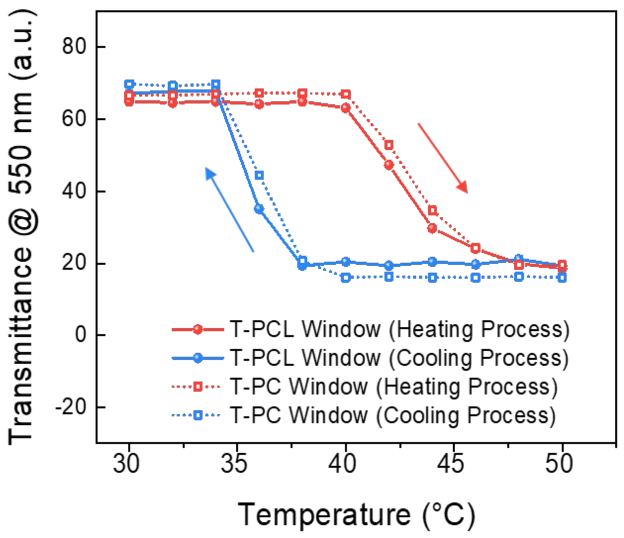

Figure S7. The optical transmittance intensity of the T-PC window and T-PCL window at 550 nm as a function of temperature upon heating and cooling processes.

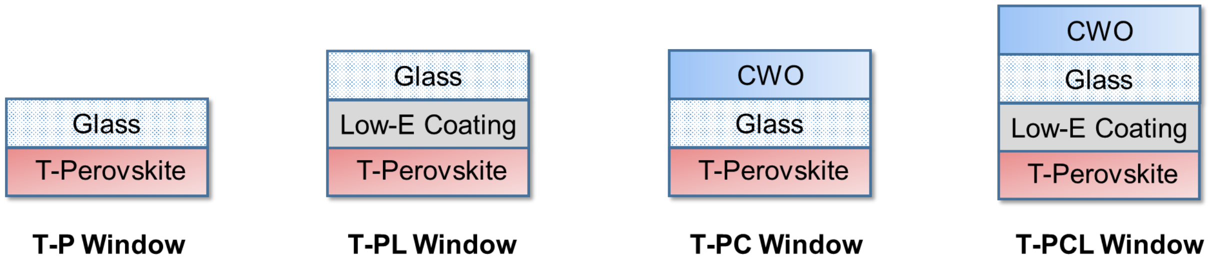

Figure S8. The schematics of T-P Window, T-PL Window, T-PC Window and T-PCL Window.

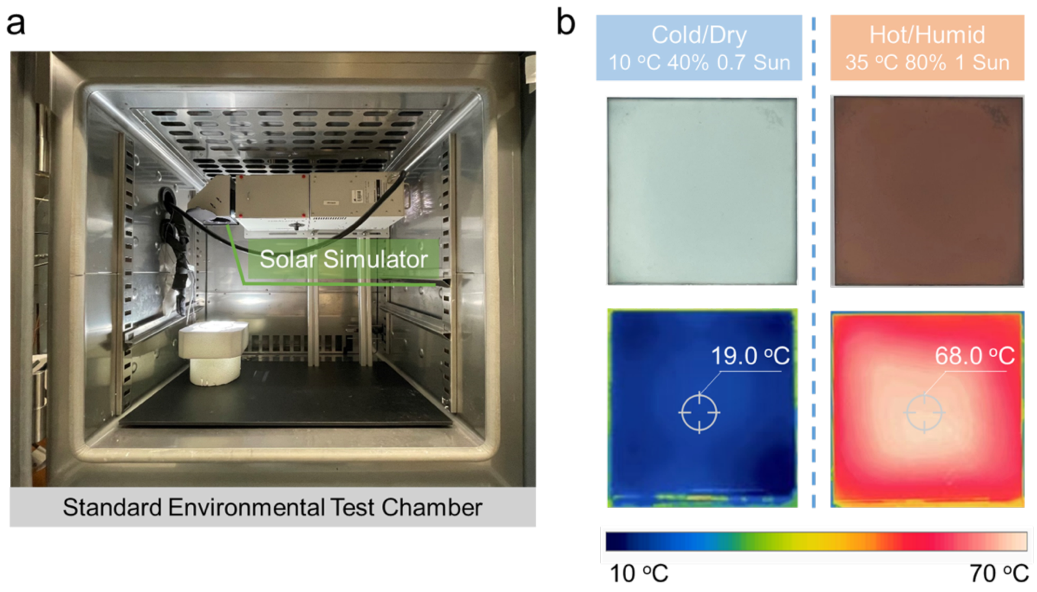

Figure S9. (a) Standard environmental test chamber to mimic different weather conditions. (b) Photos and IR images of the T-PCL window under cold/dry and hot/humid weather conditions.

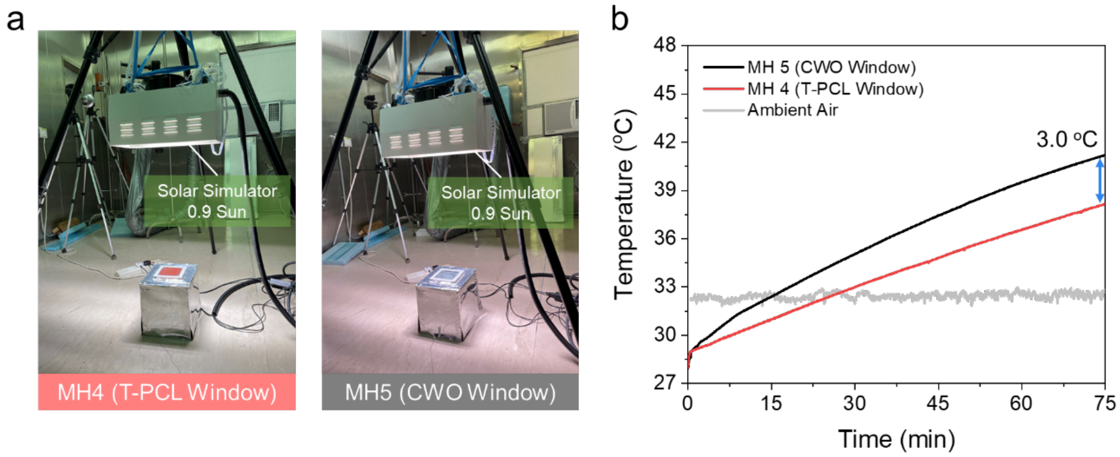

Figure S10. (a) Experimental setup for the model house experiment in the air enthalpy testing laboratory. (b) Indoor air temperature curves for the model house 4 (MH4) and model house 5 (MH5).

Supporting Note 1: The T_c of the T-Perovskite (H-$MAPbI_{3-x}Cl_x$) layer in the T-PCL window is positively associated with the humidity[1]. Therefore, an experiment was conducted to examine the thermochromism under two different weather conditions (e.g. very cold and dry as well as hot and humid weather conditions). To create the cold and dry as well as hot and humid weather conditions, the experiment was performed in a Standard Environmental Test Chamber (JEIO TECH TH-TG) fitted with a solar simulator (Figure S9a). To simulate the very cold and dry winter day, the temperature and humidity (RH) of the environmental chamber was set at 10 °C and 40%, respectively, and the power of the solar simulator set at 0.7 sun. It can be seen that in Figure S9b, with the surface temperature of the T-PCL window at only 19 °C, that the thermochromism cannot be triggered due to the cold ambient temperature and weak solar radiation. This phenomenon is desirable in cold weather since it is necessary for the smart window to remain in the transparent state to enable the sunlight to pass through the window to heat the indoor environment. Next, the environmental condition of the environmental chamber was set at 35 °C and 80% with 1 sun solar radiation to mimic the very hot and humid summer day. In this situation, the window surface temperature reached 68 °C, triggering the thermochromism and transition to the colored state due to the high ambient temperature and

strong solar absorption of the CWO layer, thereby preventing overheating of the indoor environment. This experiment proves the crucial role of the CWO layer in the T-PCL window, which helps the T-Perovskite finish the thermochromic transition under the humid and hot weather condition.

Table S1. Optical properties of normal glass, CWO glass, Low-E glass and T-Perovskite glass.

	$\tau_{lum,cold}$ (%)	$\tau_{lum,hot}$ (%)	$\Delta\tau_{sol}$ (%)	$A_{NIR,cold}$ (%)	$A_{NIR,hot}$ (%)
Normal Glass (Soda lime glass)	88.4	88.4	0	13.6	13.6
CWO Glass	74.4	74.4	0	77.8	77.8
Low-E Glass	84.4	84.4	0	28.16	28.16
T-Perovskite Glass	79.4	32.7	20.1	12.7	16.7

Table S2. Weather status of Hong Kong on Nov. 3rd, 2021 measured by the weather station

Date	Mean Air Pressure (hPa)	Minimum Air Temperature (°C)	Maximum Air Temperature (°C)	Mean Cloud Cover	Relative Humidity
Nov. 3rd, 2021	1006.9	25.6	33	65%	55.2%

Table S3. Building parameters used in the Energy Plus simulation

Building type	Large office building
Number of Floors	12
Total Floor Area	73.2 m × 48.8 m
Average Window-to-Wall Ratio	37.5%
Temperature setting point for HVAC control	Below 21 °C for heating/ Above 24 °C for cooling
Setpoint for lighting on	500 lux

Table S4. Optical information of the windows used in the simulation

	Normal Window	Low-E Window	T-PC Window		T-PCL Window	
States	-	-	Cold State	Hot State	Cold State	Hot State
Solar Transmittance	0.82	0.72	0.35	0.18	0.35	0.18

Solar Front Reflectance	0.07	0.10	0.09	0.08	0.09	0.09
Solar Back Reflectance	0.07	0.10	0.08	0.09	0.11	0.11
Visible Transmittance	0.88	0.84	0.65	0.23	0.65	0.23
Visible Front Reflectance	0.08	0.09	0.10	0.09	0.11	0.10
Visible Back Reflectance	0.08	0.09	0.10	0.10	0.11	0.10
Emissivity (Front side)	0.84	0.84	0.78	0.78	0.78	0.78
Emissivity (Back side)	0.84	0.11	0.85	0.86	0.27	0.30

Table S5. Climate information of cities used in the simulation

City (Country)	Location	Climate Types	Mean Temperature
Chengdu (China)	30°39′N, 104°03′E	**Temperate:** with relatively cold winters, and hot, humid and rainy summers.	6.5 °C -26.6 °C
Abu Dhabi (UAE)	24°28′N, 54°22′E	**Desert climate:** characterized by pleasantly mild winters and very hot, sunny summers.	17.8 °C – 35.1 °C
Hong Kong (China)	22°08′N, 113°49′E	**Subtropical:** with very mild winters and hot, rainy, and humid summers.	17.0 °C – 29.0 °C
Singapore	1°18′N, 103°51′E	**Equatorial:** hot, humid, and rainy throughout the year.	26.5 °C -28.5 °C

Reference:

[1] S. Liu, Y. W. Du, C. Y. Tso, H. H. Lee, R. Cheng, S. P. Feng, K. M. Yu, *Advanced Functional Materials* **2021**, *2010426*, 1.
